# Arsenic Trioxide and Resveratrol Show Synergistic Anti-Leukemia Activity and Neutralized Cardiotoxicity

**DOI:** 10.1371/journal.pone.0105890

**Published:** 2014-08-21

**Authors:** Yuhua Fan, Meng Chen, Jia Meng, Lei Yu, Yingfeng Tu, Lin Wan, Kun Fang, Wenliang Zhu

**Affiliations:** 1 College of Pharmacy, Harbin Medical University-Daqing, Daqing, China; 2 Department of Respiratory Medicine, the Fourth Hospital of Harbin Medical University, Harbin, China; 3 Department of Geriatrics, the Second Affiliated Hospital of Harbin Medical University, Harbin, China; 4 Department of Cardiology, the Fourth Hospital of Harbin Medical University, Harbin, China; 5 Radiology Department and Key Laboratory of Molecular Imaging, the Fourth Hospital of Harbin Medical University, Harbin, China; 6 Institute of Clinical Pharmacology, the Second Affiliated Hospital of Harbin Medical University, Harbin, China; INSERM-Université Paris-Sud, France

## Abstract

Cardiotoxicity is an aggravating side effect of many clinical antineoplastic agents such as arsenic trioxide (As_2_O_3_), which is the first-line treatment for acute promyelocytic leukemia (APL). Clinically, drug combination strategies are widely applied for complex disease management. Here, an optimized, cardiac-friendly therapeutic strategy for APL was investigated using a combination of As_2_O_3_ and genistein or resveratrol. Potential combinations were explored with respect to their effects on mitochondrial membrane potential, reactive oxygen species, superoxide dismutase activity, autophagy, and apoptosis in both NB4 cells and neonatal rat left ventricular myocytes. All experiments consistently suggested that 5 µM resveratrol remarkably alleviates As_2_O_3_-induced cardiotoxicity. To achieve an equivalent effect, a 10-fold dosage of genistein was required, thus highlighting the dose advantage of resveratrol, as poor bioavailability is a common concern for its clinical application. Co-administration of resveratrol substantially amplified the anticancer effect of As_2_O_3_ in NB4 cells. Furthermore, resveratrol exacerbated oxidative stress, mitochondrial damage, and apoptosis, thereby reflecting its full range of synergism with As_2_O_3_. Addition of 5 µM resveratrol to the single drug formula of As_2_O_3_ also further increased the expression of LC3, a marker of cellular autophagy activity, indicating an involvement of autophagy-mediated tumor cell death in the synergistic action. Our results suggest a possible application of an As_2_O_3_ and resveratrol combination to treat APL in order to achieve superior therapeutics effects and prevent cardiotoxicity.

## Introduction

Due to its substantial anticancer effect, arsenic trioxide (As_2_O_3_) has been recommended as the front-line agent for treatment of acute promyelocytic leukemia (APL), particularly for cases of relapsed or refractory APL [1]–[3]. Although generally considered a relatively safe therapeutic strategy [4], numerous clinical reports have indicated that chronic exposure to a therapeutic dose of As_2_O_3_ could damage cardiac structure and functions and evoke severe cardiac side effects such as ventricular arrhythmia, even resulting in sudden cardiac death in certain cases [5]–[8]. This issue may become increasingly relevant due to the significantly extended survival time of APL patients, and therefore increased likelihood of long-term exposure to As_2_O_3_ resulting in cardiovascular disease. Thus, prophylactic treatment is urgently required for managing the consequent cardiotoxicity in clinical applications of As_2_O_3_.

A better understanding of the potential mechanism by which As_2_O_3_ induces its cardiotoxicity will undoubtedly be of value for developing specific and effective preventive measures. Recently, many experimental observations have revealed that mitochondrial microstructural changes and dysfunctions might play crucial roles in As_2_O_3_-mediated cardiotoxicity via inducing excessive production of reactive oxygen species (ROS), and the subsequent increase in cell apoptosis [9]–[12]. Indeed, enrichment of mitochondriain cardiomyocytes enhanced their susceptibility to oxidative damage compared to other cells [13]. Accordingly, a prophylactic strategy was proposed that is based on maintaining mitochondrial function to guard against As_2_O_3_-induced oxidative stress [14]. This suggests that natural, strong antioxidants might be ideal drug candidates. Recently, such antioxidants have been investigated as rational cardioprotectants against the cardiotoxicity induced by As_2_O_3_, including the flavonoid genistein (Gen) as well resveratrol (Rev), a stilbene that is enriched in red wine [15], [16]. These investigations have pointed to the use of a combination treatment of Gen or Rev (Gen/Rev) and As_2_O_3_ as a novel therapeutic strategy for APL to prevent cardiotoxicity. Nonetheless, many important issues have yet to be considered. First, the exact mechanism regarding the cardioprotective effect of Gen/Rev against As_2_O_3_ remains elusive. Second, due to poor bioavailability of polyphenolic compounds, a reasonable and feasible choice of drugs is necessary [17]. Third, the potential antitumor effects of the use of Gen/Rev and As_2_O_3_ in combination in APL are unknown. Finally, although previous studies have validated the anticancer effect of Gen and Rev independently [18], [19], it is still unknown whether they can be effective at suppressing the proliferation of APL cancer cells and assist As_2_O_3_. This is a particularly important line of evidence that is required to determine whether the proposed new method is superior to the currently widely applied As_2_O_3_ monotherapy strategy.

Therefore, in this study, the ability of these two natural antioxidants, Gen and Rev, to reverse As_2_O_3_-induced oxidative stress injuries and simultaneously enhance the anticancer effect of As_2_O_3_ was investigated *in vitro* in neonatal rat left ventricular myocytes (NRLVMs) and NB4 cells, respectively. Our experiments focused on drug-induced alterations of mitochondria-derived ROS generation and the secondarily triggered cell apoptosis. Due to an intrinsic functional relationship between the mediators implicated in regulating oxidative stress and autophagy [20], we also measured the protein expression of LC3, a marker of cellular autophagy activity. We designed these experiments with the aim of providing mechanism-based answers to the open questions related to the potential of Gen/Rev plus As_2_O_3_ combinatorial therapy for APL.

## Materials and Methods

### Reagents and drugs

Gen and Rev were provided by Xi’an QingYue Biotechnology Co. Ltd. (China) and Sigma Chemical Co. (St. Louis, MO, USA), respectively. As_2_O_3_ was acquired from Harbin YI-DA Pharmaceutical Limited Company. The 3-(4,5-dimethylthiazol-2-yl)-2,5-diphenyl-tetrazolium bromide (MTT), cell-penetrating lipophilic cationic fluorochrome JC-1 (5,5′,6,6′-tetrachloro-1,1′,3,3′-tetraethylbenzimidazole-carbocyanide iodine), the Total Superoxide Dismutase Assay Kit with 2-(4-iodophenyl)- 3-(4-nitrophenyl)-5-(2,4-disulfophenyl)-2H-tetrazolium, monosodium salt (WST-1), and Annexin V-FITC Apoptosis Detection Kit were bought from Beyotime Institute of Biotechnology (China) and stored at −20°C in the dark. The 2′,7′-dichlorofluorescein diacetate (DCFH-DA) was provided by Molecular Probes (Eugene, OR, USA). The TUNEL detection kit was purchased from Roche (Cell Death Detection Kit; Roche Biochemicals; Mannheim, Germany). LC3A/B monoclonal antibody was purchased from Cell Signaling Technology, Inc. (Danvers, MA, USA).

### Culture of NB4 cells and NRLVMs

Human promyelocytic leukemia NB4 cell line, established in 1991 from a patient suffering from APL having the t(15;17) translocation, was a kind gift from Dr. M. Lanotte (INSERM Unit301, St Louis Hospital, Paris, France) [21]. NB4 cells were collected, washed two times in RPMI1640, counted and resuspended at 500,000 cells/ml in RPMI1640 with 10% fetal bovine serum (FBS). After 24 h cultivation, the cells were sedimented by centrifugation (1500×*g* for 5 min). NRLVMs were isolated from neonatal rat hearts of 1- to 2-day-old Sprague-Dawley rats. Briefly, the rats were immersed in 75% alcohol and decapitated, and the hearts were then quickly removed and seeded in cold Dulbecco's modified Eagle medium (DMEM). These hearts were cut into small pieces with scissors and digested with 0.25% trypsin solution. The isolated cardiomyocytes were placed in DMEM with 10% FBS and centrifuged, and pellets were resuspended and cultured for 90 min at 37°C. Cardiomyocyte-enriched suspensions were removed from the culture flask and placed in fresh medium. The use of animals complied with the Guide for the Care and Use of Laboratory Animals published by the US National Institutes of Health (NIH Publication, No. 85–23, revised 1996) and the study protocol was pre-approved by the Experimental Animal Ethics Committee of the Harbin Medical University, China (Animal Experimental Ethical Inspection Protocol, No. 2009104). NRLVMs were pretreated with Rev (5 µM) or Gen (50 µM) for 1 h and then co-incubated with As_2_O_3_ (5 µM) [22] for another 24 h. The procedure was the same for NB4 cells, except that a concentration of 2 µM As_2_O_3_ was used [23].

### Measurement of ROS production

Measurement of intracellular ROS production was based on the oxidation of DCFH-DA to fluorescent 2′,7′-dichlorofluorescin (DCF). Cells were cultured for 12 h followed by incubation with Rev (5 µM) or Gen (50 µM) for 1 h, and then co-incubation with As_2_O_3_, or were incubated with As_2_O_3_ alone for 12 h. A concentration of 5 µM and 2 µM of As_2_O_3_ was used for NRLVMs and NB4 cells, respectively. The cells were then further incubated with 10 µM DCFH-DA at 37°C for 30 min, and then washed twice with serum-free medium and stored in FBS-free medium. Cellular DCF fluorescence intensities were detected by confocal microscopy with excitation and emission spectra of 488 nm and 525 nm, respectively.

### Measurement of intracellular GSH

NB4 cells or NRLVMs were seeded in 6-well plate. After the cells grew into 90% confluence, they were treated with Rev+As_2_O_3_ or Rev at the indicated concentration. After 24 hours of Rev+As_2_O_3_ or Rev exposure, the cells were trypsinized, harvested and centrifuged at 1000×*g*, for 3 min. Cell pellets were removed to 1.5 mL eppendorf tubes, cleaned twice with cold PBS and resuspended in ice-cold metaphosphoric acid (MPA). After homogenization, the solution was centrifuged at 10,000×*g* at 4°C for 10 min and then the supernatant was applied to measure levels of GSH according to the manufacturer’s instructions (Bioxytech-GSH 400, OxisResearch, Portland, OR, USA). The assay was carried out in eppendorf tubes and transferred to flat-bottom 96-well plates for absorbance measurement at 400 nm. The pellet from the centrifugation was dissolved in 100 µL of 0.1 M NaOH and the protein concentration was determined by the Bio-Rad microprotein assay in 96-well plate using bovine serum albumin as the standard. The GSH level was expressed as nmol GSH/mg cellular protein.

### Measurement of mitochondrial membrane potential (MMP)

JC-1 was applied to explore the effects of Gen and Rev on mitochondrial function by measuring MMP in As_2_O_3_-treated cardiomyocytes and NB4 cells. Cells were placed in a 6-well plate and cultured for 12 h at 37°C and then incubated with Rev (5 µM) or Gen (50 µM) for 1 h prior to co-treatment with As_2_O_3_, or were incubated with As_2_O_3_ alone for another 12 h. A concentration of 5 µM and 2 µM of As_2_O_3_ was used for NRLVMs and NB4 cells, respectively. Red emission of the dye represents normal MMP and green fluorescence indicates mitochondria with depolarized MMP. MMP was measured using a confocal laser-scanning microscope (Fluoview-FV300; Olympus, Tokyo, Japan).

### Determination of superoxide dismutase (SOD) activity

The activity of the anti-oxidant enzyme SOD in NRLVMs and NB4 cells was detected by using a Total Superoxide Dismutase Assay Kit with WST-1 according to the manufacturer’s protocol. Briefly, cells were exposed to Rev (5 µM) or Gen (50 µM) for 1 h following treatment with As_2_O_3_ for another 24 h. A concentration of 5 µM and 2 µM of As_2_O_3_ was used for NRLVMs and NB4 cells, respectively. Then, the cell suspension was centrifuged (800×*g*, 10 min, 4°C), and the cell pellets were ultrasonicated for 15 min (every 15 s with 5-min intervals) at 4°C in cell lysate buffer [RIPA buffer, 50 mM Tris, pH 7.4, 150 mM NaCl, 1% Triton X-100, 1% sodium deoxycholate, 0.1% sodium dodecyl sulfate (SDS), sodium orthovanadate, sodium fluoride, ethylenediamine tetraacetic acid, and leupeptin]. After the cell-lysed buffer was centrifuged at 2000×*g* for 15 min, the supernatant was removed. Supernatants, enzyme-working solutions, and WST-1 were prepared and added to a 96-well plate. The mixtures were incubated at 37°C for 20 min, and the absorbance was finally determined at 450 nm using a microplate reader.

### Protein extraction and immunoblotting analysis

Protein samples were isolated from NRLVMs and NB4 cells. NRLVMs and NB4 cells were seeded in 6-well plate at 37°C in 5% CO_2_. After treatment with different types of drugs, the two types of cells were collected from 6-well plate, then the cell suspension was centrifuged (800×*g*, 10 min, 4°C), and the cell pellets were ultrasonicated for 15 min (every 15 s with 5 min intervals) at 4°C in cell lysate buffer (RIPA buffer, 50 mM Tris pH 7.4, 150 mM NaCl, 1% Triton X-100, 1% sodium deoxycholate, 0.1% SDS, sodium orthovanadate, sodium fluoride, EDTA and leupeptin). After cells-lysed buffer was centrifuged at 1000×*g* for 15 min, the supernatant protein samples were kept for the following experiments. The isolated protein samples were subjected to 15% SDS-polyacrylamide gel electrophoresis, blotted to a nitrocellulose membrane, and then blocked with 5% non-fat milk for 120 min. Next, the membranes were probed with LC3A/B in phosphate-buffered saline (PBS) containing 1% BSA and incubated overnight at 4°C. Thereafter, membranes were washed three times with PBS for 30 min and incubated with secondary antibody (Alexa Fluor; Molecular Probes; Eugene, OR, USA) for 1 h. The bands were acquired using an imaging system (LI-COR Biosciences; Lincoln, NE, USA), and quantified with Odyssey v3.0 software by measuring the band intensity [area×optical density (OD)] in each group using β-actin (anti-β-actin antibody) as an internal control for normalization.

### Measurement of cell viability

The cell viability was measured with an MTT reduction assay using a previously described method [24]. Briefly, cells were seeded in serum-free DMEM for 24 h, followed by administration with the indicated concentrations of agents at each time point. After incubation, the cells were quickly washed twice with cold PBS and added to MTT solution (final concentration, 5 mg/mL) for 4 h at 37°C. Then, the supernatant was removed and formazan crystals were dissolved with dimethylsulfoxide (150 µL) for 10 min. The absorbance was measured at 490 nm. Notably, the effect of Rev and As_2_O_3_ on the cell viability of NB4 cells was quantitatively assessed by calculating combination index (CI) as described before [25].

### TUNEL assay

The cells were treated as described above. DNA fragmentation of the cells was then determined using the TUNEL assay. Briefly, air-dried slides were fixed with 4% paraformaldehyde for 30 min at room temperature, washed three times with PBS, and then permeabilized with 1% Triton X-100 for 4 min at 4°C. Subsequently, a TdT-labeled nucleotide mix was added to each slide and incubated at 37°C for 60 min in the dark. Slides were washed twice with PBS and then counterstained with 10 mg/mL 4,6-diamidino-2-phenylindole (DAPI) for 5 min at 37°C.

### Flow cytometric analysis of cell apoptosis

The extent of apoptosis was detected by using annexinV-FITC apoptosis detection kit as described in the manufacturer’s instructions [26]. After NB4 cells or NRLVMs had been treated with Rev+As_2_O_3_ or Rev for 24 h, cells were harvested, and carefully washed with PBS for three times. After centrifugation at 1000×*g* for 5 min, the cell pellets were resuspended in 195 µL annexin V binding buffer and gently mixed by adding another 5 µL annexin V binding buffer. The suspension was then incubated in the dark for 10 min at room temperature. Thereafter, the supernatant was removed by centrifugation at 1000×*g* for 5 min. After 190 µL of annexin V binding buffer and 10 µL of propidiumiodide (50 mg/mL) were added, the fluorescence of these cells were analyzed by flow cytometry using the FloMax software. The fraction of cell population in different quadrants was analyzed using quadrant statistics. The lower left quadrant indicated normal cells; lower right quadrant represented early apoptotic cells and in the upper right quadrant was late apoptotic cells. The upper left quadrant was necrotic cells.

### Statistical analysis

Data are presented as the mean ± SEM. The significance of differences between groups was assessed using one-way ANOVA followed by Dunnett’s test. Two-tailed *p*<0.05 was considered to be a statistically significant difference.

## Results

### Co-treatment of Gen/Rev further increased As_2_O_3_-induced oxidative stress in NB4 cells but relieved oxidative stress in NRLVMs

**Figure 1:**
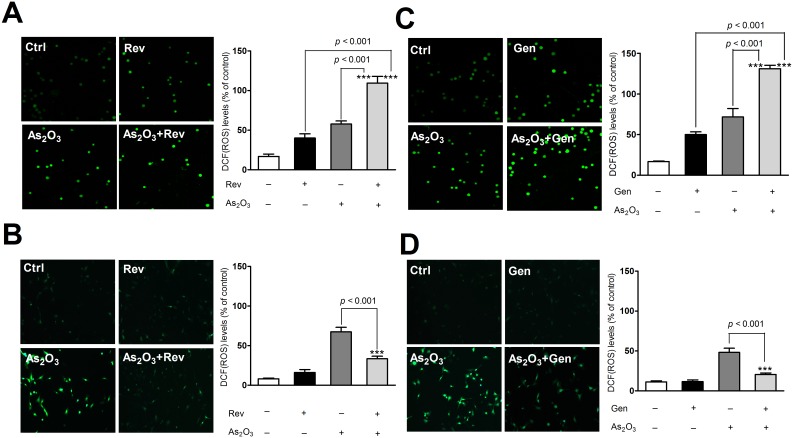
Effect of Gen/Rev on As_2_O_3_-induced oxidative stress in NB4 cells and NRLVMs (n = 6). Co-treatment of 5 µM Rev or 50 µM Gen further increased ROS production in NB4 cells compared to As_2_O_3_ alone (A, C) but reduced the ROS level in NRLVMs (B, D). ***p<0.001, As_2_O_3_+Rev versus As_2_O_3_ or Rev; ***p<0.001, As_2_O_3_+Gen versus As_2_O_3_ or Gen.

**Figure 2:**
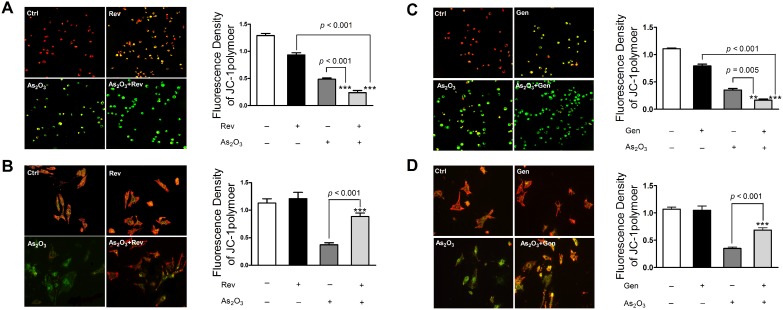
Effect of co-treatment of As_2_O_3_ and Rev/Gen on MMP of NB4 cells and NRLVMs (n = 6). Co-treatment of 5 µM Rev or 50 µM Gen further decreased the MMP in NB4 cells compared to As_2_O_3_ alone (A, C) but restored MMP in NRLVMs (B, D). ***p<0.001, As_2_O_3_+Rev versus As_2_O_3_ or Rev; ***p<0.001, As_2_O_3_+Gen versus Gen; **p = 0.005, ***p<0.001, As_2_O_3_+Gen versus As_2_O_3_.

**Figure 3:**
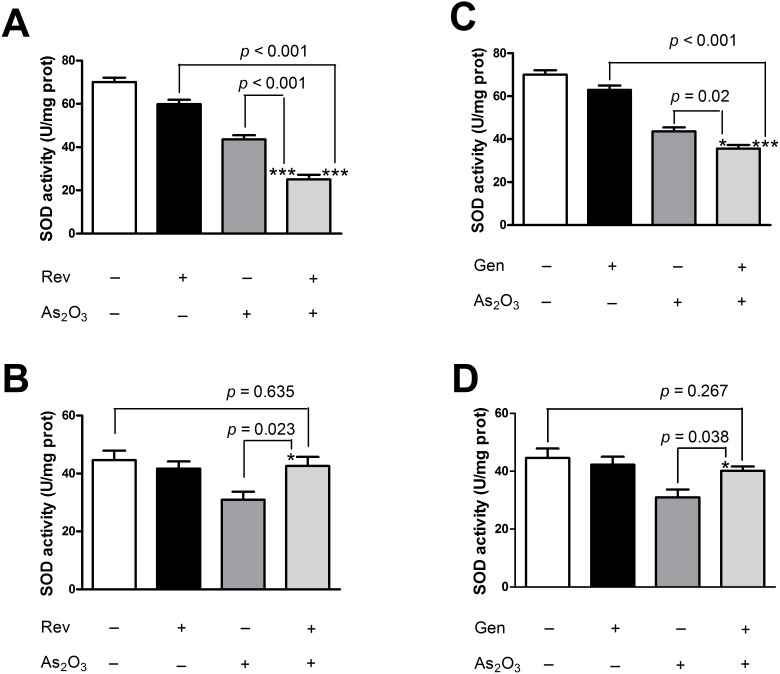
Effect of Gen/Rev on As_2_O_3_-induced SOD activity in NB4 cells and NRLVMs (n = 6). Co-treatment of 5 µM Rev or 50 µM Gen further decreased the SOD activity in NB4 cells compared to As_2_O_3_ alone (A, C) but restored it in NRLVMs (B, D). ***p<0.001, As_2_O_3_+Rev versus Rev; *p = 0.023, ***p<0.001, As_2_O_3_+Rev versus As_2_O_3_; ***p<0.001, As_2_O_3_+Gen versus Gen; *p = 0.02, **p = 0.038, As_2_O_3_+Gen versus As_2_O_3_.

Consistent with previous studies [27]–[29], the individual compounds, Rev, Gen, and As_2_O_3_, substantially induced endogenous production of ROS in NB4 cells (Figure 1A and C). Mitochondrial malfunction, in conjunction with other factors such as increased metabolic activity and oncogenic stimulation, contributed to the heightened redox status of cancer cells, whereas excessive ROS generation inevitably aggravated tumor cell damage [30]. Significant alteration of MMP clearly indicated drug-induced damage to the mitochondria, the main intrinsic source of ROS, in NB4 cells (Figure 2A and C). Combination of As_2_O_3_ and Gen/Rev led to a more dramatic release of ROS from dysfunctional mitochondria than single drug treatment. Combined application of As_2_O_3_ and Gen/Rev also caused a remarkable decline in SOD activity (Figure 3A and C). As SOD is one of the main endogenous free radical scavenging enzymes, this finding suggests the continuous accumulation of ROS. Simultaneously reduced GSH level further exacerbated the injuries by excessive cellular oxidative stress (Figure S1A). In contrast to these phenomena observed in NB4 cells, the drug combination treatment in NRLVMs showed neutralized effects on ROS generation, MMP, GSH level, and SOD activity rather than synergistic effects (Figures 1–3 and S1B). Both Rev (5 µM) and Gen (50 µM) obviously mitigated the As_2_O_3_-induced increase of ROS and mitochondrial injury in cardiomyocytes, demonstrating cytoprotection against the cardiotoxicity caused by As_2_O_3_. In addition, successful reversal of SOD activity to basal levels suggested the restored ability of cardiomyocytes to scavenge ROS.

### Gen/Rev enhanced As_2_O_3_-induced autophagy in NB4 cells and NRLVMs

**Figure 4:**
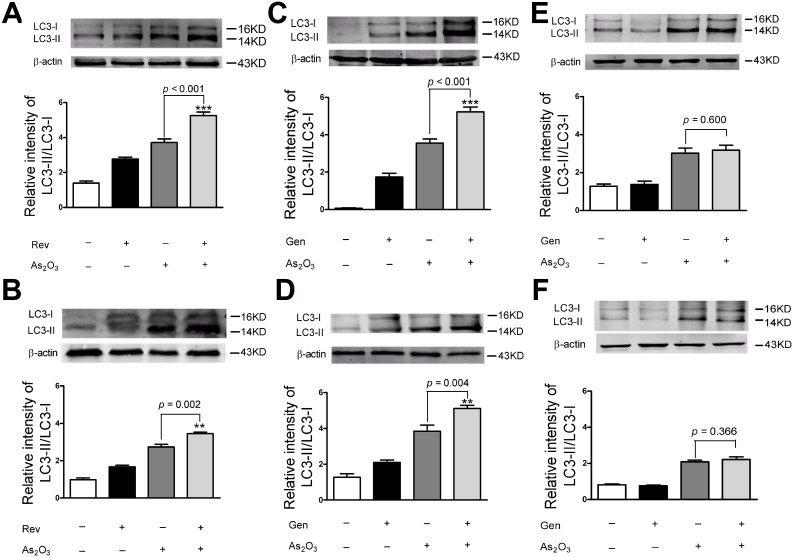
Effect of co-treatment of As_2_O_3_ and Gen/Rev on LC3 expression in NB4 cells and NRLVMs (n = 3). Co-treatment of 5 µM Rev or 50 µM Gen further increased the expression ratio of LC3 II/LC3 I in NB4 cells (A, C) and NRLVMs (B, D) compared to As_2_O_3_ alone. **p = 0.002, ***p<0.001, As_2_O_3_+Rev versus As_2_O_3_; **p = 0.004, **p<0.005, As_2_O_3_+Gen versus As_2_O_3_. No significant difference was found in the expression ratio of LC3 II/LC3 I in NB4 cells (E) and NRLVMs (F) with co-treatment of 5 µM Gen compared to As_2_O_3_ alone.

Increased release of ROS is one of the main endogenous factors for enhancement of cell autophagy [31]. Accordingly, As_2_O_3_ obviously increased the expression ratio of LC3 II/LC3 I in NB4 cells following the excessive generation of ROS (Figure 1A and 4A). Our result was in line with a previous study by Qian et al., in which another autophagy marker, Beclin-1, was confirmed to be up-regulated by As_2_O_3_ in leukemia cells [32]. Co-treatment with Rev substantially enhanced the effect of As_2_O_3_ on autophagy in NB4 cells (Figure 4A). However, to achieve the same effect with Gen, a ten-fold concentration was required (Figure 4C and E). We further confirmed that the autophagy induced by As_2_O_3_ was also enhanced by co-treatment with Gen/Rev in NRLVMs (Figure 4B and D). This result is consistent with the cardioprotection that Rev provides via activation of autophagy [33]. As observed in NB4 cells, an equal concentration of Gen failed to enhance autophagy, implying a dosage advantage of Rev relative to Gen (Figure 4F).

### Gen/Rev promoted As_2_O_3_-induced apoptosis in NB4 cells but protected against apoptosis in NRLVMs

**Figure 5:**
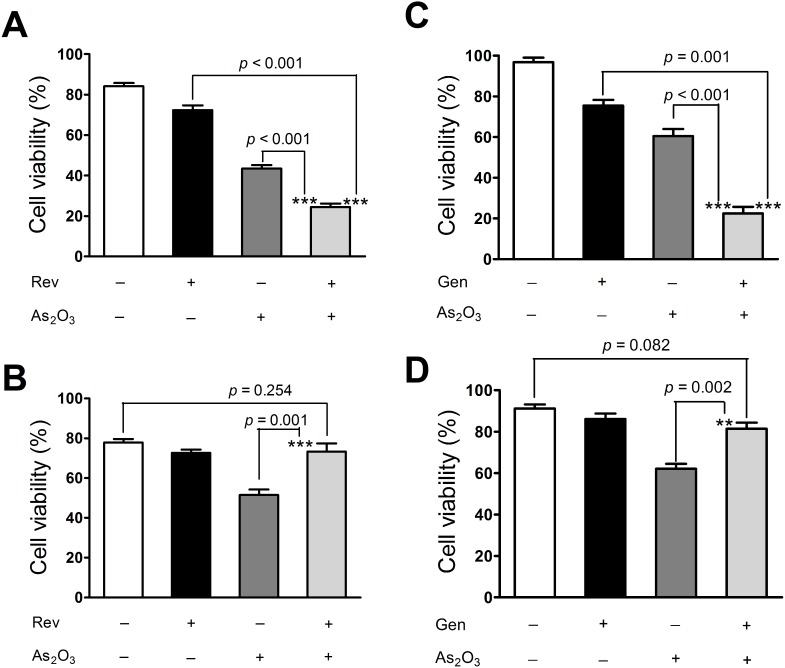
Effect of Gen/Rev on As_2_O_3_-induced cell viability of NB4 cells and NRLVMs (n = 6). Co-treatment of 5 µM Rev or 50 µM Gen further decreased the cell viability of NB4 cells (A, C) but reversed the cell viability of NRLVMs (B, D). ***p<0.001, As_2_O_3_+Rev versus Rev or As_2_O_3_; ***p<0.001, As_2_O_3_+Gen versus Gen; **p = 0.002, ***p<0.001, As_2_O_3_+Gen versus As_2_O_3_.

**Figure 6:**
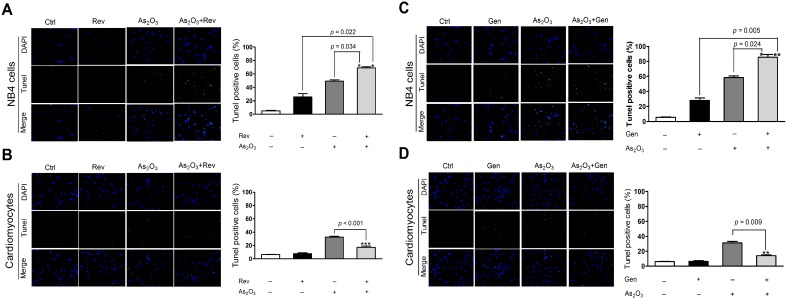
Effect of Gen/Rev on As_2_O_3_-induced apoptosis of NB4 cells and NRLVMs as determined by a TUNEL assay (n = 6). Co-treatment of 5 µM Rev or 50 µM Gen further aggravated the As_2_O_3_-induced apoptosis of NB4 cells (A, C) but reversed that of NRLVMs (B, D). ***p<0.001, As_2_O_3_+Rev versus Rev; ***p = 0.001, ***p<0.001, As_2_O_3_+Rev versus As_2_O_3_; ***p = 0.001, As_2_O_3_+Gen versus Gen; **p = 0.002, ***p<0.001, As_2_O_3_+Gen versus As_2_O_3_.

MTT and TUNEL assays consistently verified that only 2 µM As_2_O_3_ was sufficient to substantially induce cell apoptosis of NB4 cells (Figure 5A and 6A), in line with its good therapeutic effect for APL [3]. However, this apoptosis-promoting activity might also contribute to marked cardiac toxicity [16]. Our results indicated that As_2_O_3_ substantially decreased the cell viability of NRLVMs and induced cardiomyocyte apoptosis (Figure 5B and 6B). However, addition of Gen/Rev changed the picture. On the one hand, Rev or Gen further exacerbated the apoptotic damage caused by As_2_O_3_ in NB4 cells (Figure 5C and 6C), whereas no obvious damage to cell viability and apoptosis was observed in these co-treated cardiomyocytes (Figure 5D and 6D). Additionally, the results of MTT-based CI calculation indicated that Rev act synergistically with As_2_O_3_ on inducing cell apoptosis of NB4 cells (Figure S2). This finding was further confirmed by the result of flow cytometry (Figure S3A). Co-administration of 2 µM As_2_O_3_ and 5 µM Rev dramatically increased the proportions of early apoptotic cells (27.16%) and late apoptotic cells (34.82%), compared with those of the control NB4 cells (early apoptotic cells, 0.87%; late apoptotic cells, 0.73%). However, 5 µM Rev obviously alleviated As_2_O_3_-induced apoptosis in NRLVMs by substantially reducing early apoptotic cells from 20.37% to 12.17% and late apoptotic cells from 7.71% to 1.55% (Figure S3B). The above findings were consistent with the results obtained with respect to ROS generation and LC3 expression in NB4 cells and NRLVMs (Figures 1–4).

## Discussion

The outstanding benefit of As_2_O_3_ treatment for APL is due to its ability to specifically initiate the degradation of PML/RAR alpha, a core driving oncoprotein of APL [34]. Non-specific actions of As_2_O_3_, such as increasing ROS production, also greatly contribute to the mechanism by which APL can be cured with As_2_O_3_ [35]. However, as with many drugs, there is another side to these beneficial effects. The excessively amplified ROS generation flux induced by As_2_O_3_ inevitably leads to above-threshold toxicity levels in normal cells. Cardiomyocytes are likely to bear the brunt of this toxicity due to enrichment of mitochondria and their particular susceptibility to oxidative stress injury [13]. This has been validated experimentally [9]–[12] and confirmed by a plethora of clinical drug toxicity event reports [5]–[8]. In this study, combinations of As_2_O_3_ and the natural antioxidants Gen/Rev were investigated *in vitro* for the first time to explore their potential for treating APL without inducing cardiotoxicity.

Because of its multiple phenolic hydroxyl groups, the natural product Rev shows strong cytoprotective capacity against ROS generated by different inducers in non-tumor cells [36], which was confirmed in the present study. Rev successfully reversed the As_2_O_3_-induced ROS outbreak in NRLVMs. An equivalent effect was achieved with another natural antioxidant, Gen, but at a ten-fold concentration. Interestingly, we found that Rev and Gen played the role of accomplice to As_2_O_3_ in NB4 cells by exacerbating intracellular oxidative stress instead of adversary by extinguishing the ROS outbreak. Tumor cells employ a different mechanism to that of non-tumor cells for regulating mitochondrial functions [37], [38], which eventually leads to disparate effectsof the same drug in tumor cells relative to non-tumor cells. Accordingly, in this study, we validated that both Rev and Gen could exacerbate As_2_O_3_-induced mitochondrial damage in the NB4 cells, but mitigated the mitochondrial injury caused by As_2_O_3_ in cardiomyocytes, in agreement with previous studies [28], [29], [37], [38]. In addition, our experiments demonstrated that Gen/Rev further reduced SOD activity and deteriorated the intracellular ROS environment of NB4 cells by shifting the balance between ROS scavenging factors and ROS release factors. Ultimately, Gen/Rev might accelerate the As_2_O_3_-mediated degradation of PML/RARA oncoprotein via maintaining a high level of intracellular ROS, as proposed by Jeanne et al. [35]. This potential mechanism is reasonable to explain the synergistic proapoptotic effect observed by the combination of As_2_O_3_ and Gen/Rev.

While significantly relieving the oxidative injury caused by As_2_O_3_, 5 µM Rev was still able to enhance the autophagic flux of NRLVMs, indicating ROS-independent activation of autophagy. This role is likely the main contributor to Rev’s myocardial protection, as revealed in previous studies [39], [40]. Although there is currently no consensus as to whether activation or inhibition intervention of autophagy in APL is recommended [41], a study by Qian et al. strongly demonstrated that obvious enhancement of autophagy was indeed associated with the As_2_O_3_-mediated cell death of leukemia cells [32]. The results of our study further verified this finding, as autophagic cell death was implicated in the mechanisms by which As_2_O_3_ counteracts cell proliferation and promotes apoptosis of NB4 cells; Gen/Rev strengthened its pro-apoptotic effect via further elevating the level of autophagy.

In conclusion, we presented here *in vitro* evidence for synergistic antileukemic action of As_2_O_3_ and Rev from multiple aspects including oxidative stress, autophagy, and apoptosis. Meanwhile, the cardioprotective potential of Rev was also validated against As_2_O_3_-induced cardiomyocytes injury. Compared with Gen, the lower effective concentration of Rev indicates its potential as a rational drug candidate for APL treatment in combination with As_2_O_3_. Our findings provide a novel therapeutic possibility for APL with enhanced efficiency and reduced toxicity. Further functional experiments *in vivo* are required to validate our findings.

## Supporting Information

Figure S1 **Co-treatment of As_2_O_3_ and Rev on total GSH level in NB4 cells and NRLVMs (n = 6).** Co-treatment of 5 µM Rev further decreased the GSH level in NB4 cells (A) but reversed that of NRLVMs (B). ***p<0.001 or *p* = 0.001, versus As_2_O_3_+Rev.
(TIF)

Figure S2 **Result of CI calculation of the combinations of As_2_O_3_ and Rev on the cell viability of NB4 cells (n = 6).** A. Effect of As_2_O_3_+Rev combinations of different concentrations on the cell viability of NB4 cells. B. CI values of As_2_O_3_+Rev combinations at different concentrations. A CI value of less than 1 means synergistic action by As_2_O_3_ and Rev.
(TIF)

Figure S3 **Result of flow cytometric analysis of cell apoptosis in NB4 cells and NRLVMs.** Co-treatment of 5 µM Rev and 2 µM As_2_O_3_ synergistically promoted early and late apoptosis instead of necrosis in NB4 cells (**A**). Addition of 5 µM Rev markedly relieved cardiomyocyte apoptosis that was induced by 5 µM As_2_O_3_ (**B**).
(TIF)
