# Supplementary figures and images for: Arsenic Trioxide and Resveratrol Show Synergistic Anti-Leukemia Activity and Neutralized Cardiotoxicity
Source: PLoS One. 2014 Aug 21;9(8):e105890. doi: 10.1371/journal.pone.0105890 (PMC4140836; doi:10.1371/journal.pone.0105890)

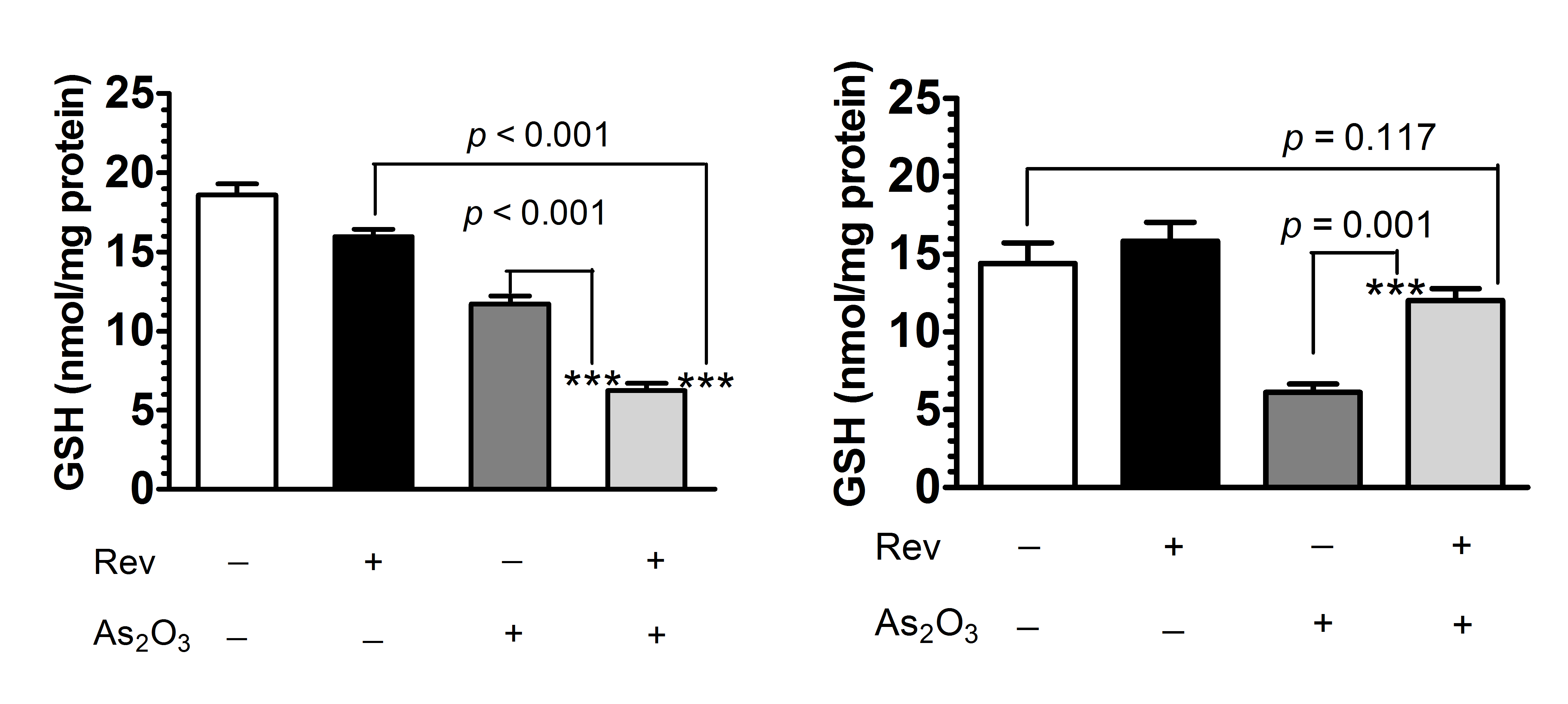

Supplement: Figure S1 — Co-treatment of As2O3 and Rev on total GSH level in NB4 cells and NRLVMs (n = 6). Co-treatment of 5 µM Rev further decreased the GSH level in NB4 cells (A) but reversed that of NRLVMs (B). ***p<0.001 or p = 0.001, versus As2O3+Rev. (TIF) [file pone.0105890.s001.tif]

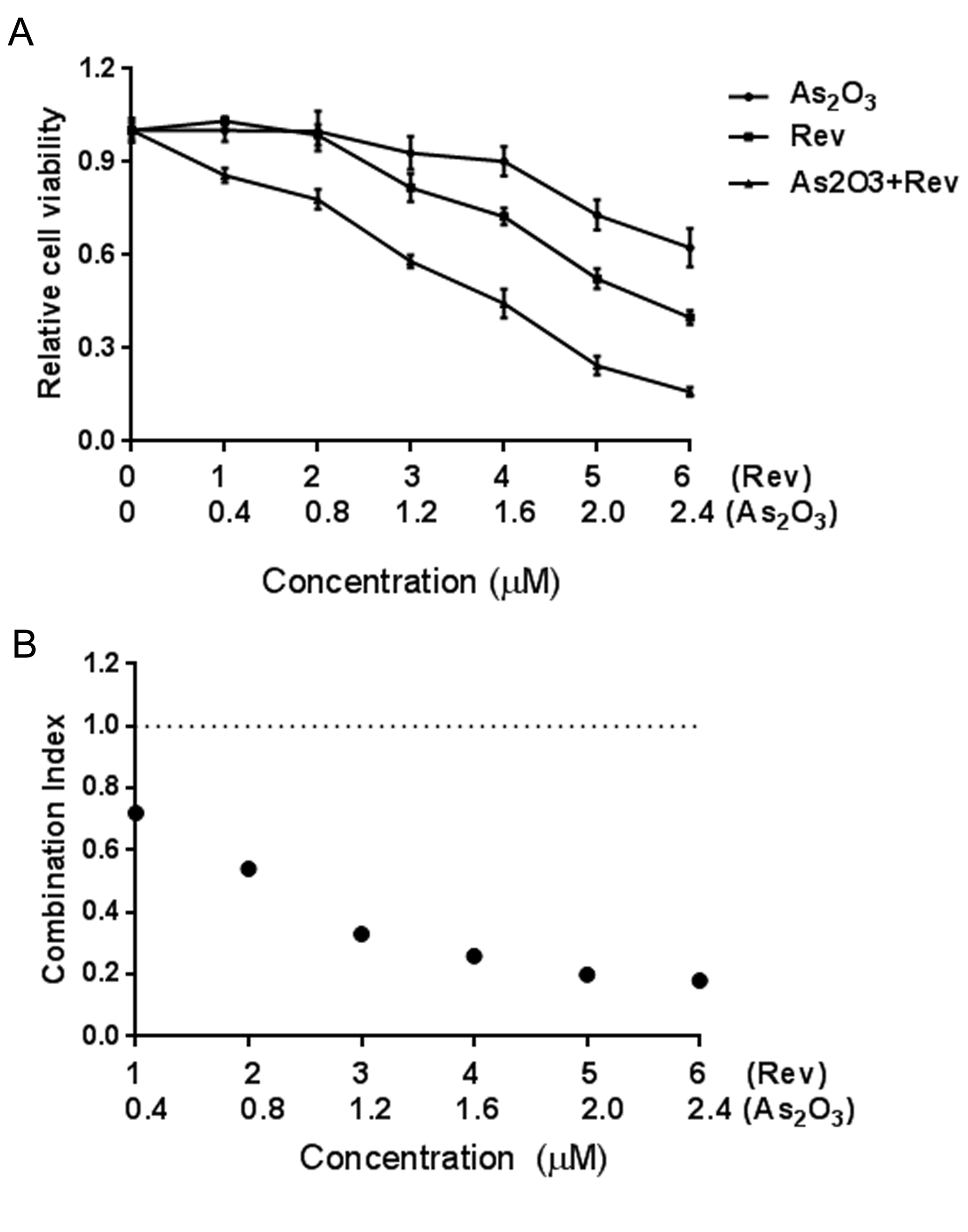

Supplement: Figure S2 — Result of CI calculation of the combinations of As2O3 and Rev on the cell viability of NB4 cells (n = 6). A. Effect of As2O3+Rev combinations of different concentrations on the cell viability of NB4 cells. B. CI values of As2O3+Rev combinations at different concentrations. A CI value of less than 1 means synergistic action by As2O3 and Rev. (TIF) [file pone.0105890.s002.tif]

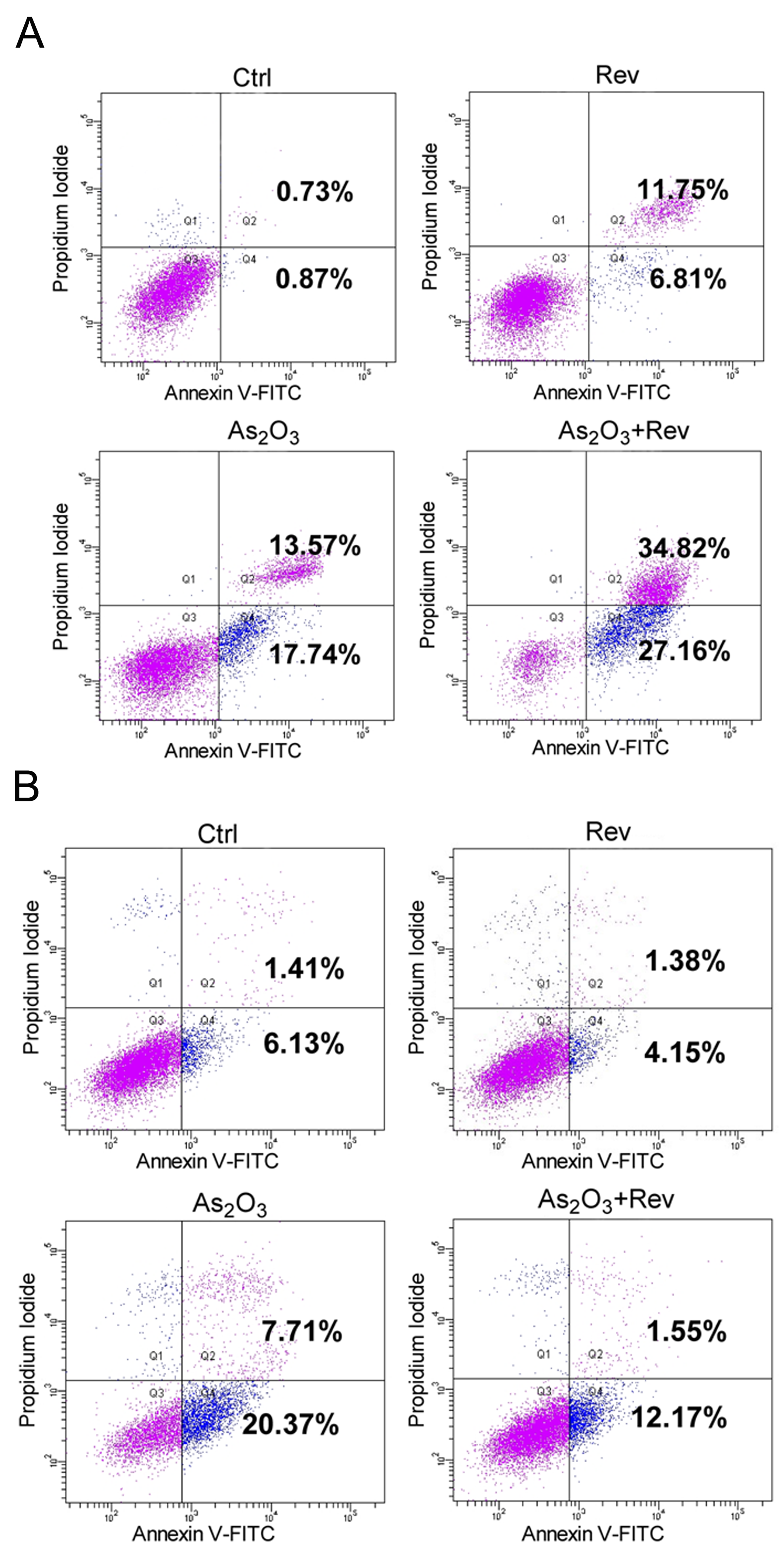

Supplement: Figure S3 — Result of flow cytometric analysis of cell apoptosis in NB4 cells and NRLVMs. Co-treatment of 5 µM Rev and 2 µM As2O3 synergistically promoted early and late apoptosis instead of necrosis in NB4 cells (A). Addition of 5 µM Rev markedly relieved cardiomyocyte apoptosis that was induced by 5 µM As2O3 (B). (TIF) [file pone.0105890.s003.tif]
